# *Cannabis sativa* demonstrates anti-hepatocellular carcinoma potentials in animal model: in silico and in vivo studies of the involvement of Akt

**DOI:** 10.1186/s42238-023-00190-z

**Published:** 2023-07-12

**Authors:** Dorcas I. Akinloye, Damilohun S. Metibemu, Mujidat T. Shittu, Mariam A. Lawal, Faith O. Olatunji, Muideen A. Oyediran, Oluseyi A. Akinloye

**Affiliations:** 1 Department of Biochemistry, Phyto-chemistry and Bio-computing Research Laboratory, College of Biosciences, Federal University of Agriculture, Abeokuta, Nigeria; 2 Department of Chemistry, Physics, and Atmospheric Sciences, Jackson State University, Jackson, MS 39217-0095 USA

**Keywords:** Anti-angiogenic, Proapoptotic, Anti-inflammatory, Δ-9-tetrahydrocannabinol, Cannabidiol

## Abstract

**Background:**

Targeting protein kinase B (Akt) and its downstream signaling proteins are promising options in designing novel and potent drug candidates against hepatocellular carcinoma (HCC). The present study explores the anti-HCC potentials of *Cannabis sativa* (*C. sativa*) extract via the involvement of Akt using both in silico and in vivo animal models of HCC approaches.

**Methods:**

Phytoconstituents of *C. sativa* extract obtained from Gas Chromatography Mass-spectrometry (GCSM) were docked into the catalytic domain of Akt-2. The Diethylnitrosamine (DEN) model of HCC was treated with *C. sativa* extract. The effects of *C. sativa* extract treatments on DEN model of hepatocellular carcinoma were assessed by One-way analysis of variance (ANOVA) of the treated and untreated groups

**Result:**

The lead phytoconstituents of *C. sativa* extract, Δ-9-tetrahydrocannabinol (Δ-9-THC) and cannabidiol form stable hydrophobic and hydrogen bond interactions within the catalytic domain of Akt-2. *C. sativa* extract (15 mg/kg and 30 mg/kg) respectively gives a 3-fold decrease in the activities of liver function enzymes when compared with the positive control (group 2). It also gives a 1.5-fold decrease in hepatic lipid peroxidation and elevates serum antioxidant enzymes’ activities by 1-fold in HCC treated Wistar rats when compared with the positive control (group 2). In an animal model of hepatocellular carcinoma*, C. sativa* extract significantly downregulated Akt and HIF mRNAs in groups 3, 4, and 5 with 2, 1.5, 2.5-fold decrease relative to group 2. VEGF mRNA was downregulated by 1.5-fold decrease in groups 3-5 when compared to group 2. The expression of XIAP mRNA was downregulated by 1.5, 2, and 1.25-folds in groups 3, 4, and 5 respectively, in comparison with group 2. In comparison to group 2, COX-2 mRNA levels were downregulated by 1.5, 1, and 1-folds in groups 3–5. In groups 3–5, CRP mRNA was downregulated by 2-fold in comparison with group 2. In groups 3–5, p21 mRNA was upregulated by 2, 2.5, and 3-folds, respectively when compared with group 2. It upregulated p53 mRNA by 2.5, 3.5, and 2.5-folds in groups 3–5 in comparison with group 2. It downregulated AFP mRNA by 3.5, 2.5, .2.5-folds in groups 3, 4, and 5 respectively when compared with group 2. Histologic analysis showed that *C. sativa* extract reduced necrosis and inflammation in HCC.

**Conclusion:**

*C. sativa* demonstrates anti-hepatocellular carcinoma potentials in an animal model of HCC and with the involvement of Akt. Its anticancer potential is mediated through antiangiogenic, proapoptotic, cycle arrest, and anti-inflammatory mechanisms.

In future studies, the mechanisms of anti-HCC effects of Δ-9-tetrahydrocannabinol (Δ-9- THC) and cannabidiol via the PI3K-Akt signaling pathways should be explored.

## Background

Hepatocellular carcinoma (HCC) represents over 85% of primary liver cancers. Liver cancers cause about one-fourth of cancer-related mortality (Yang et al. 2019). According to WHO, over 1 million patients will lose their lives to liver cancer in 2030 (World Health Organization 2020). Liver cancer represents the second most lethal neoplasm after pancreatic cancer (Jemal et al. 2004). The incidence of HCC is high in patients with underlying liver disease, mostly due to hepatitis B or C virus (HBV or HCV) infection or alcohol abuse. However, nonalcoholic fatty liver disease (NAFLD), metabolic syndrome, and obesity are increasing the incidence of liver cancer in western nations (Younossi et al. 2019). Liver cancer is most prevalent in Africa and the far East (Franca et al. 2004). In the current state of HCC public health, there is no approved drug for its prevention (Villanueva 2019). Sorafenib, an oral multikinase inhibitor approved in 2007, is under a lot of scrutiny due to its perceived reduced median survival of less than 10 months (Sanoff et al. 2016). For advanced HCC, multiple drug candidates are now available including lenvatinib, regorafenib, cabozantinib, ramucirumab, and nivolumab. However, their short overall survival (OS) end points and their associated side effects (such as seizures, dermatitis, gastrointestinal bleeding, hypertension, pneumonitis, and stomatitis) make them unsuitable for clinical trials.

Targeting the protein kinase B (Akt) and its downstream signaling proteins are promising options in designing novel and potent drug candidates against HCC (Dimri and Satyanarayana 2020). Akt, a serine/threonine kinase is at the crossroad of neoplasm development (Dimri and Satyanarayana 2020). Natural products have been reported to inhibit the PI3K/Akt/mTOR signaling (Huang 2013). *C. sativa* is also known as hemp or marijuana, it is a specie of the Cannabinaceae family of the plant and have been shown to demonstrate anticancer potentials (Massi et al. 2013, Velasco et al. 2016, Tomco et al. 2020). As far back as the mid-1970s, Munson et al. (1975) discovered that Tetrahydrocannabinol (THC), Δ^8^-THC and cannabinol inhibit Lewis lung adenocarcinoma growth in mice. In 2006, a pilot clinical trial involving nine glioblastoma patients found that intracranial administration of THC was safe for the treatment of cancer (Guzman et al. 2006). Glioma cells were the most commonly used cellular model in studies of *C. sativa*’s anticancer effects in the 2000s (Hinz and Ramer 2022). However, a broad range of tumor cell lines from various entities have been examined over time. Numerous mechanisms by which cannabinoids inhibit tumorigenesis have indeed been outlined. Among these, protein kinase B (Akt) inhibition proves to be an essential mechanism. However, there is a paucity of data on cannabinoids' anti-tumoral effects on hepatocellular carcinoma via Akt inhibition.

The present study hereby explores the anticancer potentials of *C. sativa* extract via the involvement of Akt using both in silico and in vivo animal model of HCC approaches.

## Methodology

### Extraction and gas chromatography mass-spectrometry (GCMS) of *C. sativa*

Dried *C. sativa* https://powo.science.kew.org/taxon/urn:lsid:ipni.org:names:306087-2 leaves were obtained with permission from the National Drug Law Enforcement Agency (NDLEA), Headquarters, Abeokuta, Nigeria. Dr. O. A. Obembe, a plant taxonomist at Adekunle Ajasin University, Akungba-Akoko, Nigeria, authenticated the *C. sativa* leaves with voucher number 195. A voucher specimen was later deposited at the Herbarium of Adekunle Ajasin University (AAUAH). The *C. sativa* leaves were pulverized into powder. Three hundred and seventy-seven grams (377 g) of the pulverized *C. sativa* leaves were soaked in 2.5 L of petroleum ether for 48 hours and filtered (Romano and Hazekamp, 2013). The concentration of the filtrate was carried using a rotary evaporator. The GCMS analysis was as earlier reported by Akinloye et al. (2021).

### Docking of phytoconstituents against the Akt

The phytoconstituents of *C. sativa* extract obtained from the GCMS analysis above were retrieved from PubChem. The Akt (PDB, 3E88 and 2.5 Aº) protein receptor was downloaded from the protein databank (http://rcsb.org). *C. sativa* extract’s phytoconstituents were docked into catalytic domain of 3E88 using the grid coordinate (*X* = − 23.38, *Y* = 48.05, *Z* = − 7.43) of the co-crystallized, aminofurazan compound. Autodock 4.0 was employed for the docking. Molecules of water within the catalytic domain of 3E88 were removed. Validation of the docking scores was carried out by docking the co-crystallized ligand back into the catalytic site of 3E88 and estimate the root mean square deviation of the redocked ligand and the co-crystallized.

### Wistar rats

Thirty (30) male Wistar rats between 150 and 180 g were obtained from the Institute of Advanced Medical Research and Training (IAMRAT), College of Medicine, University of Ibadan, Nigeria. They were handled based on the recommendation of the Code of Ethics of the World Medical Association (Declaration of Helsinki). The experimental protocols were endorsed by the Institutional Animal Ethics Committee (IAEC), with ethical approval number, FUNAABIEC/19/03.

### Experimental design

The Wistar rats were grouped with respect to their weights into 5 groups of 6 Wistar rats per group.

Group 1, were given Wistar rats food and water only. Groups 2–4, were administered intra-peritoneally with 200 mg/kg of Diethylnitrosamine (DEN) and 0.5 mL/kg of carbon tetrachloride (CCl_4_) once a week for 3 weeks consecutively (Akinloye et al. 2021). Groups 3–4, were treated p.o (Per os): oral administration) for 3 weeks with 15 mg/kg and 30 mg/kg of *C. sativa* extract respectively. Group 5 did not receive DEN but were treated concurrently with groups 3–4 with 30 mg/kg of *C. sativa* extract. The animals were sacrificed through cervical dislocation on week 15 following DEN induction.

Note: The treatment commenced week 12 after the HCC had been confirmed histologically.

This was carried out by removing the liver of a randomly selected Wistar rat. The liver was preserved in buffered formalin at 10% for 24 h. With running water, the fixative was washed away. The liver tissue was dehydrated, washed in methyl-benzoate, and then set in paraffin wax. Hematoxylin and eosin were used to stain the liver sections after it had been cut into 3–5-μ-thick pieces. Light microscopy observations were made of the sections.

### Liver function assays

The activities of aspartate aminotransferase (AST), alanine aminotransferase (ALT), alkaline phosphatase (ALP), and lactate dehydrogenase (LDH) were evaluated in the serum of the Wistar rats. These were carried out using Randox Kits from Randox Laboratories Ltd. (55 Diamond Road,

Crumlin, County Antrim, BT29 4QY, United Kingdom Ardmore, United Kingdom). The protocols as stated in the manufacturer’s (Randox Laboratories Ltd.) manuals were used.

### Evaluation of superoxide dismutase and catalase activities

The activities of superoxide dismutase and catalase were evaluated in the serum. These were carried following the methods reported by Misra and Fridovich, (1972) and Hadwan and Abed (2016) respectively.

#### Evaluation of lipid peroxidation

The methods described by Farombi et al. (2000) was used to measure the formation of thiobarbituric acid reactive substances (TBARS).

### Reverse transcription-polymerase chain reaction

RNAs isolation from the liver, reverse transcription, and quantitative PCR were carried out as earlier reported by Akinloye et al. (2021). The primers are as follows (Table 1):

**Table 1:** The nucleotide sequences of the primers used

Target gene	Forward 5’-3’	Reverse 5′-3′
GAPDH	AAGGGCTCATGACCACAGTC	GGATGCAGGGATGATGTTCT
COX 2	GATGACGAGCGACTGTTCCA	TGGTAACCGCTCAGGTGTTG
IL 6	GACTTCCAGCCAGTTGCCTT	GCAGTGGCTGTCAACAACAT
CRP	GCAGTAGGTGGGCCTGAAAT	CCCGTCAAGCCAAAGCTCTA
P21	GAGAACTGGGGAGGGCTTTC	TCCTGAGCCTGTTTCGTGTC
VEGR	CGGGCCTCTGAAACCATGAA	GCTTTCTGCTCCCCTTCTGT
AKT 2	ACAGACTGTGCCCTGTCCAC	CGTGGCCTCCAGGTCTTGAT
MMP-2	AGAGGATACCCCAAGCCACT	AAAGGCAGCGTCTACTTGCT
P27	GACTCACTCGCGGCTCC	GGCTCCCGTTAGACACTCTC
AFP	CACCATCGAGCTCGGCTATT	TCCCAAAAACTCGCTTGGGT
P53	CCCCTGAAGACTGGATAACTGT	TCTCCTGACTCAGAGGGAGC
BAX	AGGACGCATCCACCAAGAAG	CAGTTGAAGTTGCCGTCTGC
XIAP	TGCATAATGAGGACTGGGGG	GCCCCGATCAGGAAAAACAC
BAD	CTTGAGGAAGTCCGATCCCG	GCTCACTCGGCTCAAACTCT

### Histopathological studies

The Histological analysis of the livers were carried as earlier reported by Akinloye et al. (2021). The livers were kept in 10% buffered formalin for 24 h. The fixative was washed away using running water. The liver tissues were dehydrated, washed in methyl-benzoate, and then embedded in paraffin wax. The liver sections were stained with hematoxylin and eosin after being cut into 3–5-mm-thick pieces. The sections were examined using light microscopy.

### Statistical analysis

GraphPad Prism 7 was used for the one-way analysis of variance (ANOVA). Mean ± standard error of the mean (SEM) was used to express data from groups 1 to 5. The *P* < 0.05 was set as the significance difference between the groups.

Note: GAPDH (glyceraldehyde-3-phosphate dehydrogenase) and mRNA levels (gene expression) were quantified using ImageJ software. The mean and standard error of the mean (SEM) of each gel image were obtained from ImageJ. For each group, the control (GAPDH) and the gene expression were normalized (normalization (Gene/GAPDH) × 100) and their respective SEM were also normalized (normalization (SEM of the gene/GAPDH SEM), that is the error bar on each histogram plot). Prism version 7 was used for plotting the graphs of relative mRNA expression (mean values, *Y* axis) against the groups (*X* axis) and the ANOVA analysis.

## Results

### Δ-9- THC, cannabidiol, and cannabinol as the major components of *C. sativa*

**Fig. 1:**
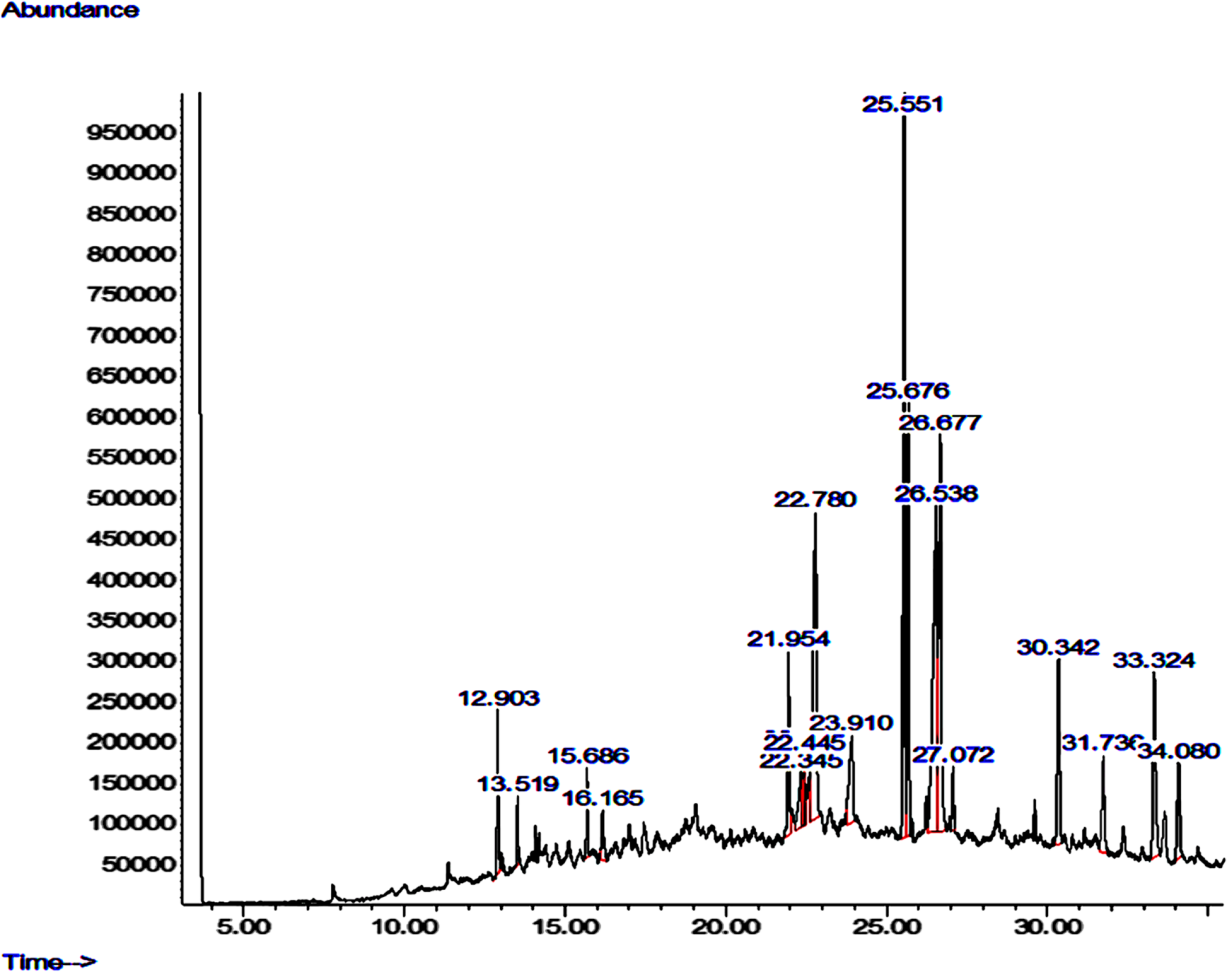
GC-MS chromatogram of *C. sativa* extract

Figure 1 shows the GC-MS chromatogram of the phytoconstituents of the *C. sativa* extract. Δ-9-tetrahydrocannabinol (Δ-9-THC), cannabidiol, and cannabinol with percentage composition of 16.26%, 14.98%, and 13.486% respectively are the major components of the extract (Table 2).

**Table 2:** Gas-chromatography-mass spectrometry profiles of *C. sativa* extract

Name	Retention time	Height	Area	Total
Caryophyllene	12.903	199885	5469672	2.421%
Humulene	13.519	84897	2273244	1.006%
Caryophyllene oxide	15.686	108877	2851290	1.262%
Octadecanal	16.165	60722	2707189	1.198%
Pentadecane	21.954	222716	7159469	3.169%
17-Pentatriacontene	22.345	66952	4090780	1.811%
2-pentadecanone	22.417	92705	3880932	1.718%
Phytol	22.445	85790	2238384	0.991%
Cannabinol	22.780	375981	30466852	13.486%
Eicosanoic acid	23.910	106439	8271611	3.661%
Δ-9-tetrahydrocannabinol	25.551	1018978	36738929	16.263%
Caryophyllenol	25.676	532074	17820294	7.888%
Cannabidiol	26.538	396198	33834639	14.977%
Oleic acid	26.677	484266	28258524	12.509%
Humulene oxide	27.072	77698	2848796	1.261%
Methanone	30.342	227246	10497238	4.647%
Nerolidol	31.736	118114	6901660	3.055%
Cannabichromene	33.324	226087	13132800	5.813%
Cannabicoumaronone	34.080	116888	6469606	2.864%

There are 19 phytoconstituents in the *C. sativa* extract, Δ-9-tetrahydrocannabinol, cannabinol, and cannabidiol constitute 16.263%, 13.486%, and 14.977% respectively of the extract

### Phytoconstituents of *C. sativa* extract demonstrate inhibition of Akt

**Fig. 2:**
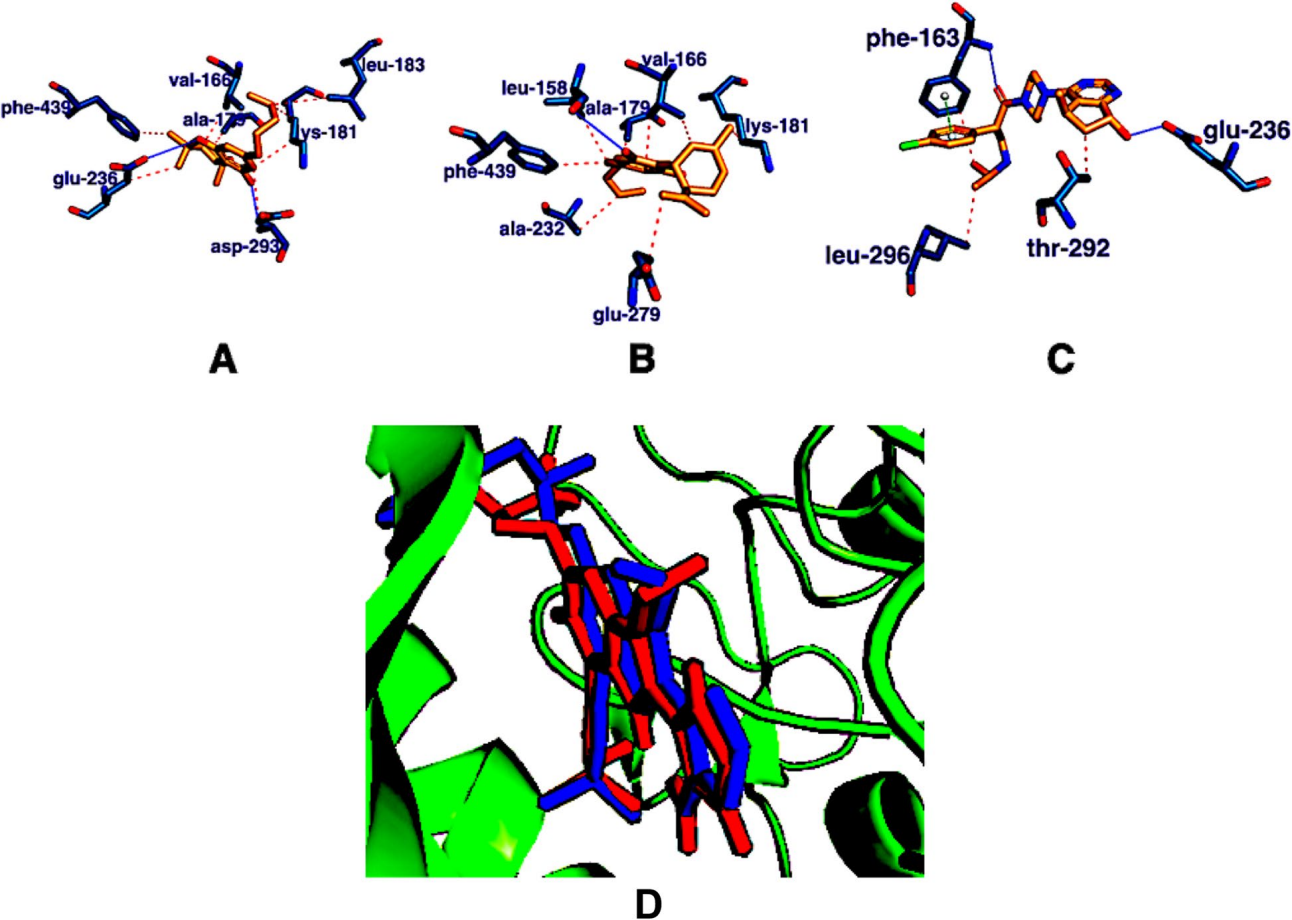
3E88 in combination with **A** THC (orange), **B** cannabidiol (orange), and **C** ipatasertib (orange). The blue lines show hydrogen bond interactions, the red lines depict hydrophobic interactions, and the green line depicts pi stacking interaction. **D** The binding poses of the re-docked (blue) and the co-crystallized compound (red) within the catalytic domain of 3E88

Docking of the phytoconstituents of *C. sativa* extract against the catalytic domain of Akt-2 shows they are potent inhibitor of Akt (Table 3). Two of the major components of the *C. sativa* extract, THC and cannabidiol possess the highest binding affinities with Akt-2, with binding energies of -10.3 kcal/mol and -10.0 kcal/mol respectively (Table 3). The lead compounds THC and cannabidiol demonstrate better inhibition of Akt when compared with ipatasertib (standard drug) with a binding energy of − 9.8 kcal/mol. Ipatasertib forms two hydrogen bond interactions (glu-236, phe-163), two hydrophobic interactions (leu-296, thr-292), and one pie stacking interaction (phe-163), THC forms seven hydrophobic interactions (phe-439, glu-236, val-166, ala-179, lys-181, leu-183, asp-293) and two hydrogen bonds (glu-236, asp-293), while cannabidiol form seven hydrophobic interactions (phe-439, leu-158, ala-179, val-166, lys-181, glu-279, ala-232) and one hydrogen bond (Fig. 2).

**Table 3:** Virtual high throughput screening of the phytoconstituents of *C. sativa* extract against the catalytic domain of Akt-2

S/N	*C. sativa*	Docking score (kcal/mol)
1	Δ-9-tetrahydrocannabinol	− 10.3
2	Caryophyllene oxide	− 10.0
3	Cannabidiol	− 10.0
4	Caryophyllenol	− 9.9
5	Caryophyllene	− 9.8
6	Cannabicoumaronone	− 9.6
7	Humulene	− 9.5
8	Humulene oxide	− 9.3
9	Cannabichromene	− 8.9
10	Cannabinol	− 8.2
11	Pentadecane	− 7.5
12	Nerolidol	− 7.1
13	phytol	− 6.9
14	Oleic acid	− 6.3
15	17-pentatriacontene	− 6.1
16	Eicosanoic acid	− 6.0
17	2-pentadecanone	− 5.9
18	Octadecanal	− 5.8
19	Methanone	− 5.5

### Validation of docking scores

Validating docking studies requires re-docking of the co-crystallized ligand into the catalytic site of the protein. When the co-crystallized ligand was re-docked into the catalytic site of 3E88, a deviation of 0.301 Å was observed (Fig. 2D). The docking is considered accurate and reliable when the deviation is less than 2.0 Å (Morris et al. 1998).

### *C. sativa* extract attenuated liver enzymes’ activities

**Fig. 3:**
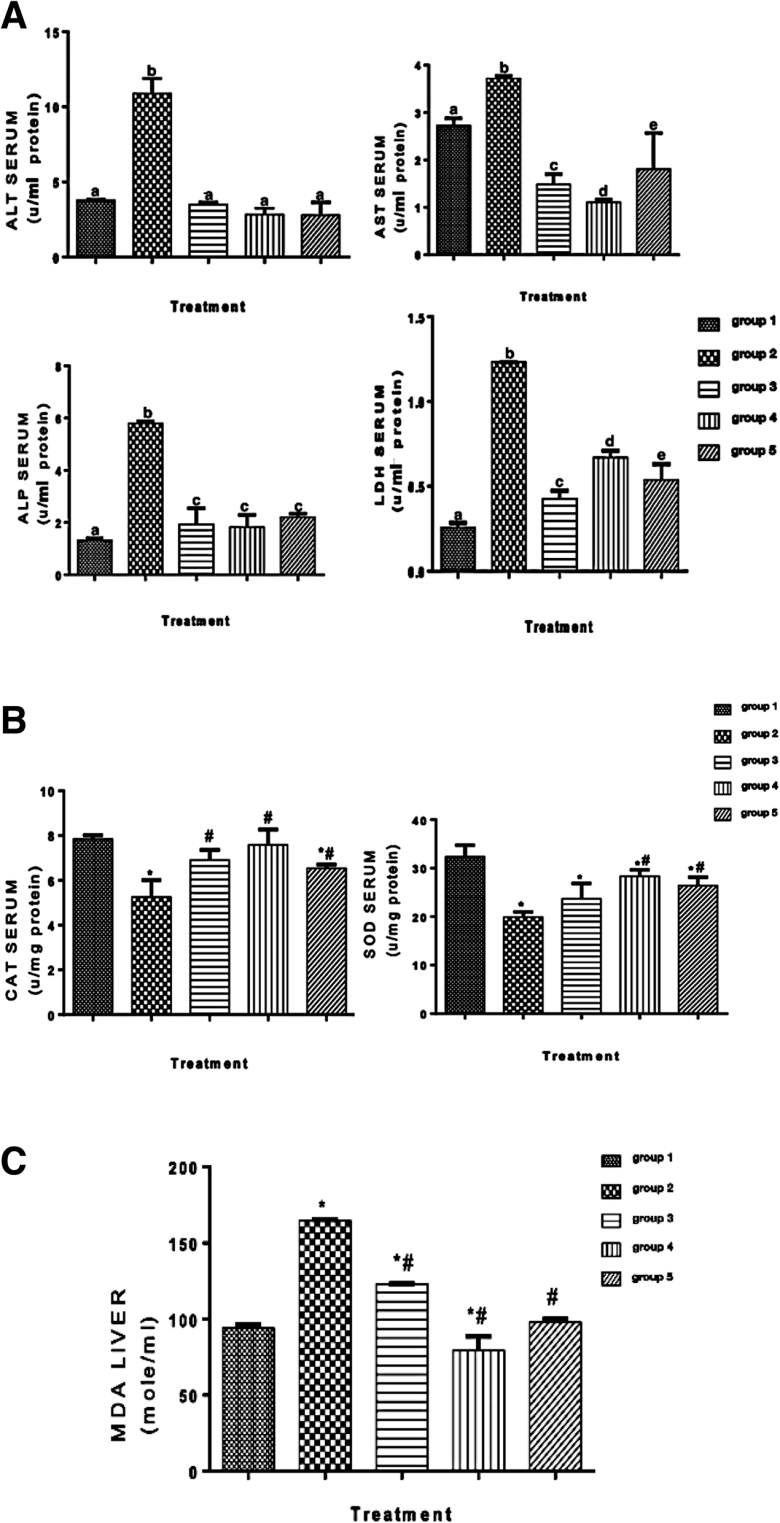
**A** Activities of liver function enzymes, ALT, AS, ALP, and LDH. Different letters on the bars show significant different. Significance is at *p* < 0.05. Significance is at *p* < 0.05. **B** Catalase and superoxide dismutase activities in the serum. *Significant difference when groups 2–5 are compared with group 1, # significant difference when groups 3–5 are compared with group 2. Significance is at *p* < 0.05. **C** MDA concentration in the liver. *Significant difference when groups 2–5 are compared with group 1, # significant difference when groups 3–5 are compared with group 2. Significance is at *p* < 0.05

It has been reported that liver enzyme activities play a significant role in the prognosis of HCC (Zhang et al. 2017). In Fig. 3A, the activities of liver functions enzymes (AST, ALT, ALP, and LDH) were significantly attenuated by treating with 15 mg/kg and 30 mg/kg of *C. sativa* extract (groups 3–5) when compared with the untreated group (group 2).

### *C. sativa* extract increase the activities of antioxidant enzymes

High oxidative stress and low antioxidant capacities are believed to play a significant role in the development and progression of hepatocellular carcinoma (HCC) (Hsiao et al. 2021).

Catalase and superoxide dismutase activities were significantly increased by treating with 15 mg/kg and 30 mg/kg of *C. sativa* extract (groups 3-5) when compared with the untreated group (group 2) (Fig. 3B).

### *C. sativa* extract ameliorates lipid peroxidation in HCC

Chronic necroinflammation of the liver leads to lipid peroxidation and oxidative stress, which contribute to hepatocellular carcinoma (HCC). (Feng et al. 2021). The concentration of malondialdehyde (MDA) (lipid peroxidation marker) in the liver was attenuated significantly by treating with 15 mg/kg and 30 mg/kg of *C. sativa* extract (groups 3–5) when compared with the untreated group (group 2) (Fig. 3C). The concentration of MDA was upregulated significantly in the group that received DEN alone (group 2) when compared with group 1.

### *C. sativa* extract downregulates Akt mRNA

**Fig. 4:**
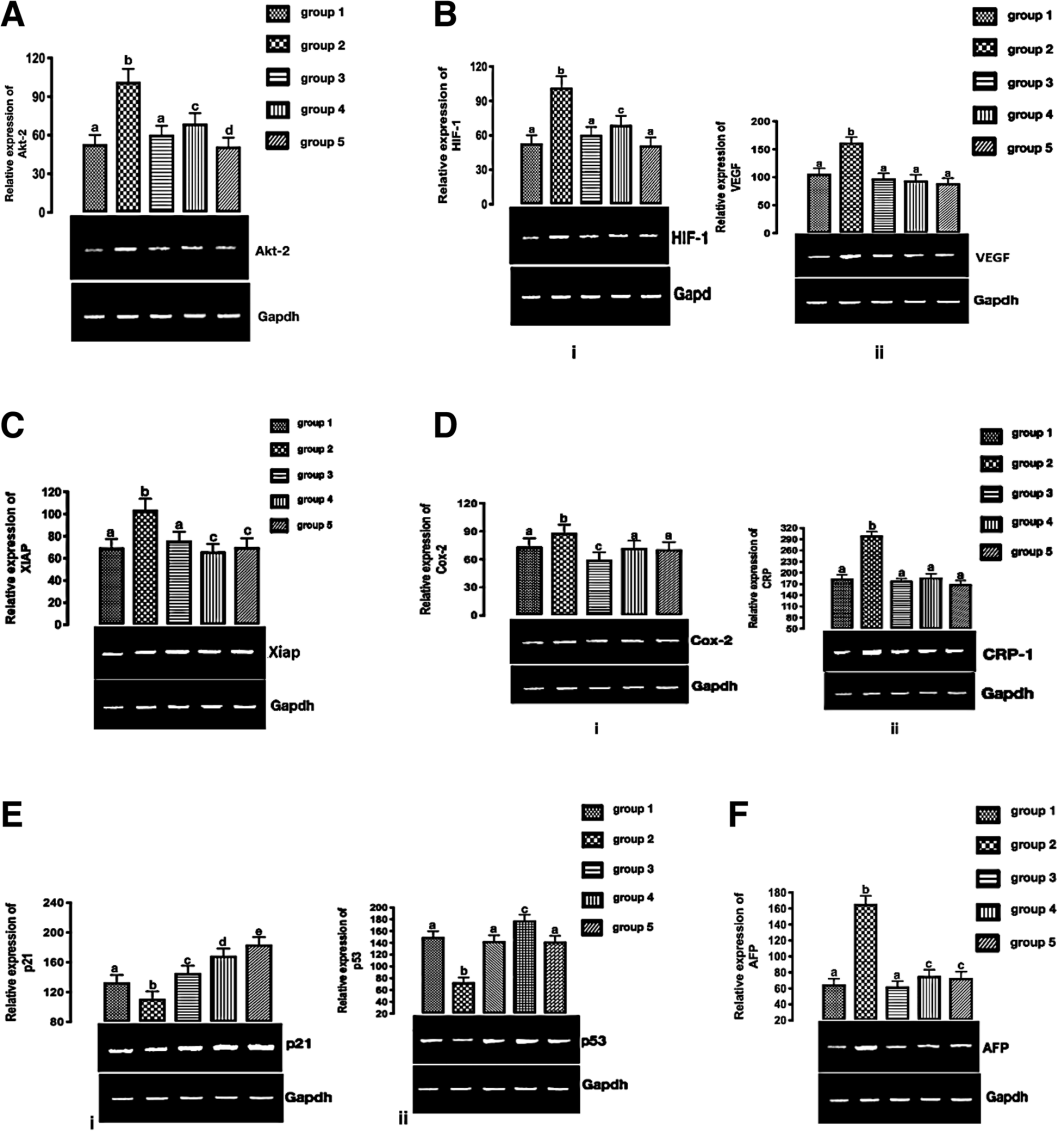
**A** Expression of Akt-2 mRNA in HCC. Different letters on the bars show significant different. Significance is at *p* < 0.05. **B** (i) HIF-1 and (ii) VEGF mRNAs expression in HCC. Different letters on the bars show significant different. Significance is at *p* < 0.05. **C** Expression of XIAP mRNA in HCC. Different letters on the bars show significant different. Significance is at *p* < 0.05. **D** COX-2 and (ii) CRP mRNAs expression in HCC. Different letters on the bars show significant different. **E** (i) p21 and (ii) p53 mRNAs expression. Different letters on the bars show significant different. Significance is at *p* < 0.05. **F** AFP mRNA expression. Different letters on the bars show significant different. Significance is at *p* < 0.05

According to Tang et al. (2018), upregulation and activation of the Akt play a role in HCC invasion and metastasis. Direct and indirect anti-tumor activity can be observed when Akt is inhibited (Mroweh et al. 2021). Akt mRNA was upregulated significantly in the group that received DEN alone (group 2) when compared with group 1. The expression of Akt mRNA was significantly increased by treating with 15 mg/kg and 30 mg/kg of *C. sativa* extract (groups 3–5) when compared with the untreated group (group 2) (Fig. 4A).

### *C. sativa* extract downregulate Akt signaling cascade mRNAs

The expression patterns of mRNAs are associated with the signaling effects of Akt (angiogenesis, apoptosis, inflammation, and evasion of cell cycle arrest) were determined.

#### *C. sativa* extract downregulates angiogenic-related mRNAs

The hypoxia-inducible transcription factors (HIFs) regulate cellular metabolism, angiogenesis, proliferation, and migration, enabling a cell to respond to hypoxia (Wilson et al. 2014) and HCC is marked by overexpression of VEGF, which is considered the force driving physiological and pathological angiogenesis (ElGhandour et al. 2021). HIF and VEGF mRNAs were significantly upregulated in the group that received DEN alone (group 2) when compared with group 1. The expression of HIF-1 and VEGF mRNAs were significantly downregulated by treating with 15 mg/kg and 30 mg/kg of *C. sativa* extract (groups 3–5) when compared with the untreated group (group 2) (Fig. 4B).

#### *C. sativa* extract downregulate X-linked inhibitor of apoptosis protein (XIAP) mRNA

The overexpression of the X-linked inhibitor of apoptosis (XIAP) protein in hepatocellular carcinoma promotes metastasis and tumor recurrence (Shi et al. 2008).

XIAP mRNA was significantly upregulated in the group that received DEN alone (group 2) when compared with group 1. However, treating with 15 mg/kg and 30 mg/kg of *C. sativa* extract (groups 3–5) significantly downregulate XIAP mRNA when compared with the untreated group (group 2) (Fig. 4C).

#### *C. sativa* extract downregulate pro-inflammatory mRNAs

Carcinogenesis has been linked to cyclooxygenase-2 (COX-2) (Bae et al. 2001) and the COX-2 gene is upregulated in HCC (Chen et al. 2017).

The expression of pro-inflammatory cyclooxygenase-2 (COX-2) and C-reactive protein (CRP) mRNAs were significantly upregulated in the group that received DEN alone (group 2) when compared with group 1. They were significantly downregulated by treating with 15 mg/kg and 30 mg/kg of *C. sativa* extract (groups 3–5) when compared with the untreated group (group 2)

(Fig. 4D).

#### *C. sativa* extract upregulate cell cycle arrest mRNAs

Cell cycle arrest and apoptosis are triggered by increased p21 and p53 transcriptional activities (Engeland 2022).

P21 and p53 mRNAs expression were significantly upregulated in the group that received DEN alone (group 2) when compared with group 1. On the other hand, p21 and p53 mRNAs expression were significantly downregulated by treating with 15 mg/kg and 30 mg/kg of *C. sativa* extract (groups 3–5) when compared with the untreated group (group 2) (Fig. 4E).

### *C. sativa* extract downregulate AFP mRNA expression

The only tumor biomarker routinely used in the treatment of hepatocellular carcinoma is alpha-fetoprotein (AFP) (HCC). AFP levels are strongly linked to tumor aggressiveness. Its concentrations are linked to poorly differentiated HCC, tumor size, and microvascular invasion. (Muscari and Maulat 2020).

AFP mRNA expression was significantly upregulated in the group that received DEN alone (group 2) when compared with group 1. However, AFP mRNA expression was significantly downregulated by treating with 15 mg/kg and 30 mg/kg of *C. sativa* extract (groups 3–5) when compared with the untreated group (group 2) (Fig. 4F).

### *C. sativa* extract moderate liver necrosis in HCC

**Fig. 5:**
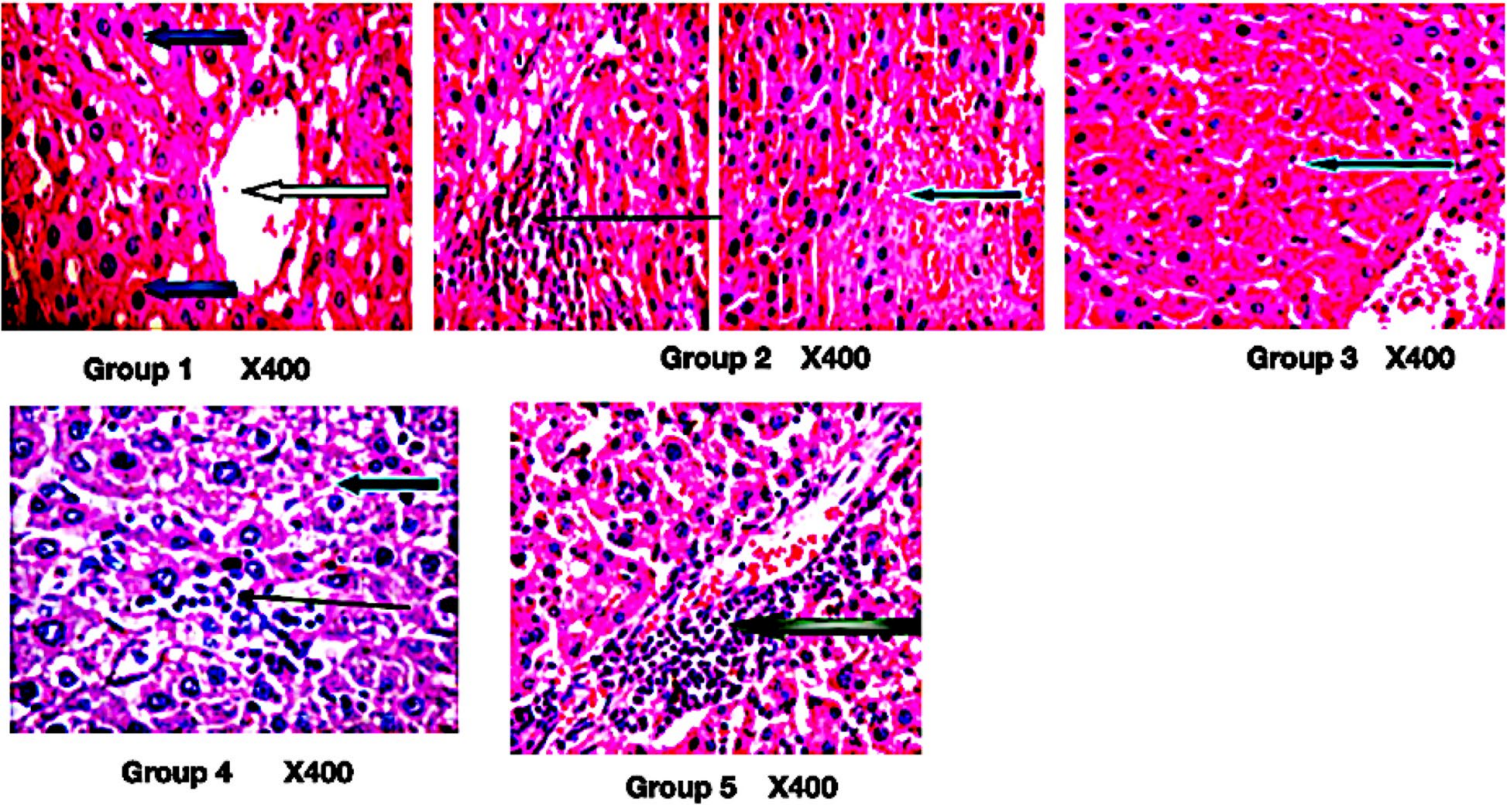
Photomicrograph of liver section stained by hematoxylin and eosin. Group 1: normal architecture as seen in lower magnification, the central venules appears mildly crowded (white arrows), the sinusoids appear normal (slender arrow) and devoid invasion of inflammatory cells, the hepatocytes revealed morphology that are normal (blue arrow). Group 2: mild vascular congestion (white arrow), there is a focal area of hepatocytes with necrosis (blue arrow) and infiltration of inflammatory cells (slender arrow). Group 3: focal area of moderate necrosis (blue arrow) and mild infiltration of inflammatory cells. Group 4: hepatocytes with fat infiltration (severe macovesicular and necrosis (blue arrow), there is mild portal triditis (white arrow), and sinusoid show mild infiltration (slender arrow). Group 5: portal infiltration of portal triads (black arrow), hepatocytes appear normal (blue arrow), and sinusoids show no infiltration (slender arrow)

*C. sativa* extract moderate necrosis and reduce inflammation in HCC (Fig. 5).

## Discussion

Liver cancer represents the second most deadly neoplasm (Jemal et al. 2004, Yang et al. 2019). In this study, we showed that *C. sativa* demonstrates anti-hepatocellular carcinoma potentials in animal model and with the involvement of Akt, a protein that is central to neoplastic development. GCMS analysis of the *C. sativa* extract revealed delta-9-tetrahydrocannabinol (Δ-9-THC), cannabidiol, and cannabinol with percentage composition of 16.26%, 14.98%, and 13.486% respectively as the major components of the extract. Molecular docking of the phytoconstituents of *C. sativa* extract against Akt-2 showed they inhibit Akt-2, a promising alternative in the design of novel and potent drug candidates against HCC. THC and cannabidiol possess the highest binding affinities with Akt-2 and also demonstrate better inhibition than ipatasertib. The better inhibition showed when compared to ipatasertib may not be unconnected with the formation of seven hydrophobic interactions and two hydrogen bonds within the Akt-2 catalytic domain by THC and seven hydrophobic interactions and one hydrogen bond by cannabidiol when compared with ipatasertib with two hydrogen bond interactions, two hydrophobic interactions, and one pie stacking interaction. The inhibition of Ak-2 by phytoconstituents of the *C. sativa* extract as shown by the molecular docking studies herein confirmed the report of Ozaita et al. (2007) that THC increased the phosphorylation of Ak-2. The RMSD of 0.301 Å showed by re-docking the co-crystallized ligand into the active domain of 3E88 further confirmed the validity of the in silico results (Morris et al. 1998).

The activities of liver enzymes are predictive and prognostic of HCC (Zhang et al. 2017). Increased activities of liver function enzymes are characteristics of HCC (Lala et al. 2020), as shown herein. However, treatment of HCC with 15 mg/kg and 30 mg/kg of *C. sativa* extract (groups 3-5) significantly attenuated the activities of liver function enzymes in group 3-5, when compared with group 2. The attenuation of the activities of the liver function enzymes demonstrated herein corroborated the reported hepatoprotective potentials of *C. sativa* (Ismail et al. 2018, Stohs and Ray 2020).

The significant decrease shown in the activities of catalase and superoxide dismutase enzymes in group 2 is in tandem with the report of Cheng et al. (2017) that HCC patients have increased oxidative stress and reduced antioxidant enzyme. However, treatment with *C. sativa* extract (groups 3–5) significantly increase the activities of these enzyme. This may be as a result of the reported antioxidant potentials of *C. sativa* (Girgih et al. 2011).

Upregulation and activation of the Akt have been implicated in the invasion and metastasis of HCC (Tang et al. 2018). In this study, treatment with *C. sativa* extract (groups 3–5) significantly downregulated Akt mRNA expression when compared with group 2. Inhibition of Akt as shown in the in silico studies may account for this observation. This also corroborated the report of Ellert-Miklaszewska et al. (2005) that cannabinoids down-regulate PI3K/Akt and Erk signaling. It also confirmed the report of Vara et al. (2011) that carotenoids inhibit the serine-threonine kinase Atk/mammalian target of rapamycin C.

Anti-angiogenic mRNAs HIF-1 and VEGF expression were downregulated significantly with *C. sativa* extract treatments (groups 3–5). This depicts that the *C. sativa* extract possesses anti-angiogenic potentials and hitherto confirm the inhibition of Akt, since Akt is known to be at the crossroad of angiogenesis (Dimri and Satyanarayana 2020). The downregulation of the anti-angiogenic mRNAs by the *C. sativa* extract further confirmed the reported anti-angiogenic properties of *C. sativa* (Solinas et al. 2012).

The upregulation of p21 and p53 mRNAs and hitherto downregulation of XIAP mRNA by the *C. sativa* extract treatments (groups 3–5), further corroborated the report of Ellert-Miklaszewska et al. (2005) that cannabinoid-induced inhibition of Akt leads to apoptosis and cell cycle arrest.

The downregulation of the pro-inflammatory mRNAs (COX-2 and CRP) by the *C. sativa* extract treatments (groups 3–5) corroborated its reported anti-inflammatory potentials (Nagarkatti et al. 2009) that *C. sativa* demonstrated anti-inflammatory potentials. Also, the downregulation of CRP and COX-2 further gives credence to the anti-HCC potentials of the *C. sativa* extract because high serum level of CRP has been reported to signify poor prognosis of patients with HCC (Zheng et al. 2013), while COX-2 overexpression has been shown to inhibit tumor cell apoptosis (Wang et al. 2019).

Alpha-fetoprotein (AFP) is still the main biomarker of liver neoplasm (Chan et al. 2017). AFP mRNA expression was upregulated in the group that received DEN alone (group 2) when compared with other groups. Furthermore, treatment with *C. sativa* extract (groups 3–5) significantly downregulated AFP mRNA. This shows treatment with *C. sativa* extract (groups 3–5) possess anti-HCC potentials.

Necrosis and inflammation are the hallmarks of HCC (Chan et al. 2017, Yu et al. 2018). The amelioration of necrosis and inflammation by the *C. sativa* extract further confirmed its anti-HCC potentials.

## Conclusion

We established that *C. sativa* demonstrates anti-hepatocellular carcinoma potentials in an animal model of HCC and with the involvement of Akt. THC and cannabidiol form stable hydrophobic and hydrogen bond interactions within the catalytic domain of Akt-2. *C. sativa* extract reduced the activities of liver function enzymes. It ameliorates lipid peroxidation and increases the antioxidant enzymes’ activities. It shows anti-angiogenic, proapoptotic, and anti-inflammatory effects. It also demonstrates cell cycle arrest. *C. sativa* extract further demonstrates its anti-HCC effects by moderating necrosis and reduce inflammation in HCC. In future studies, the mechanisms of anti-HCC effects of Δ-9-tetrahydrocannabinol (Δ-9- THC) and cannabidiol via the PI3K-Akt signaling pathways should be explored. Although preclinical trials have demonstrated the clinical efficacy of *C. sativa*, clinical trials with cancer patients are lacking. It is imperative to review the results of prospective and randomized studies on the use of *C. sativa* in cancer treatment before drawing any conclusions.

## Data Availability

The supporting data are available, contact the corresponding author.

## Abbreviations

HCC: Hepatocellular carcinoma
Akt: Protein kinase B
GCSM: Gas Chromatography Mass-spectrometry
DEN: Diethylnitrosamine
Δ-9-THC: Δ-9-tetrahydrocannabinol
WHO: World Health Organization
HBV or HCV: Hepatitis B or C virus
NAFLD: Nonalcoholic fatty liver disease
OS: Overall survival
NDLEA: National Drug Law Enforcement Agency
IAMRAT: Institute of Advanced Medical Research and Training
AST: Aspartate aminotransferase
ALT: Alanine aminotransferase
ALP: Alkaline phosphatase
LDH: Lactate dehydrogenase
TBARS: Thiobarbituric acid reactive substances
RNA: Ribonucleic acid
ANOVA: One-way analysis of variance
SEM: Standard error of the mean
HIF-1: Hypoxia-inducible factor-1
VEGF: Vascular endothelial growth factor
XIAP: X-linked inhibitor of apoptosis protein
COX-2: Cyclooxygenase-2
CRP: C-reactive protein
AFP: Alpha-fetoprotein
H and E: Hematoxylin and eosin
